# Arginase Signalling as a Key Player in Chronic Wound Pathophysiology and Healing

**DOI:** 10.3389/fmolb.2021.773866

**Published:** 2021-10-29

**Authors:** Denis C. Szondi, Jason K. Wong, Leah A. Vardy, Sheena M. Cruickshank

**Affiliations:** ^1^ Lydia Becker Institute of Immunology and Inflammation, Manchester Academic Health Science Centre, Faculty of Biology, Medicine and Health, University of Manchester, Manchester, United Kingdom; ^2^ Blond McIndoe Laboratories, Division of Cell Matrix Biology and Regenerative Medicine, Manchester Academic Health Science Centre, School of Biological Sciences, Faculty of Biology, Medicine and Health, The University of Manchester, Manchester, United Kingdom; ^3^ Skin Research Institute of Singapore, A*STAR, Singapore, Singapore

**Keywords:** wound healing, arginase (ARG), chronic wounds, polyamines, diabetic foot ulcers (DFU), venous leg ulcers (VLU)

## Abstract

Arginase (ARG) represents an important evolutionarily conserved enzyme that is expressed by multiple cell types in the skin. Arg acts as the mediator of the last step of the urea cycle, thus providing protection against excessive ammonia under homeostatic conditions through the production of L-ornithine and urea. L-ornithine represents the intersection point between the ARG-dependent pathways and the urea cycle, therefore contributing to cell detoxification, proliferation and collagen production. The ARG pathways help balance pro- and anti-inflammatory responses in the context of wound healing. However, local and systemic dysfunctionalities of the ARG pathways have been shown to contribute to the hindrance of the healing process and the occurrence of chronic wounds. This review discusses the functions of ARG in macrophages and fibroblasts while detailing the deleterious implications of a malfunctioning ARG enzyme in chronic skin conditions such as leg ulcers. The review also highlights how ARG links with the microbiota and how this impacts on infected chronic wounds. Lastly, the review depicts chronic wound treatments targeting the ARG pathway, alongside future diagnosis and treatment perspectives.

## Introduction

The skin represents a multifaceted organ with a complex architecture and biology. Due to the vast array of skin components and functions, it follows that cutaneous wound healing has to be a well-coordinated cascade of intricate events to ensure a re-establishment of protection against environmental hazards and infections.

**GRAPHICAL ABSTRACT:**
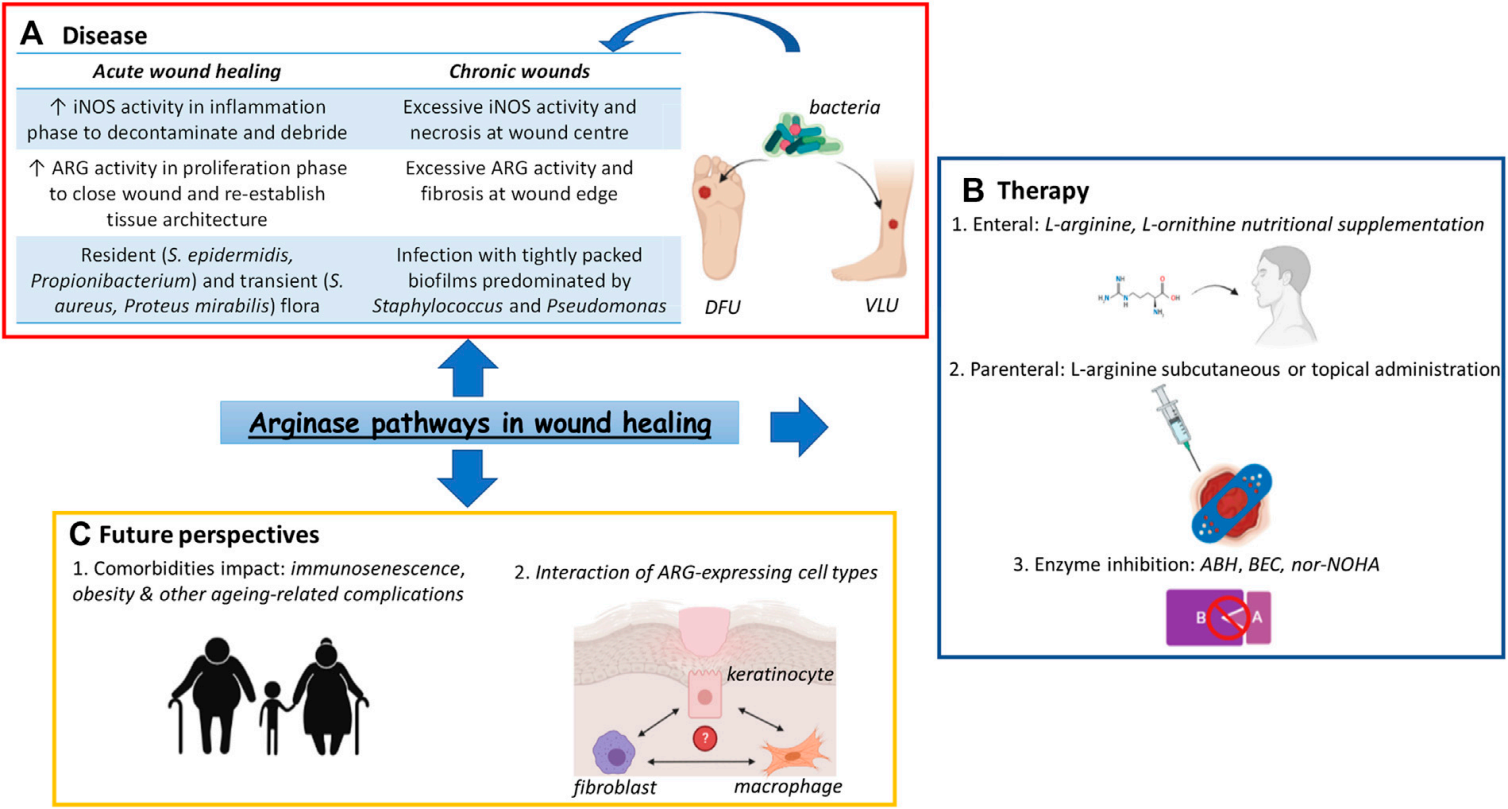
Arginase (ARG) pathways in wound healing. **(A)** Dysfunctionalities of ARG pathways have been regarded as contributing factors to the occurrence of chronic wounds such as DFUs or VLUs, the presence of bacterial populations adding a further layer of complexity to the pathophysiology of these conditions **(B)** Various treatment options based on the activity of the ARG pathways have been devised, popular choices being amino acid supplementation by both enteral and parenteral routes as well as enzyme inhibitors, such as the potent drug nor-NOHA. **(C)** However, ARG pathways-based monotherapies have showed limited success, highlighting the need to focus on patient comorbidities as well as ARG-expressing cell interactions when devising future chronic wound treatments. Abbreviations: ARG, arginase; ABH, 2(S)-amino-6-boronohexanoic acid; BEC, S-(2-boronoethyl)-L-cysteine; DFU, Diabetic Foot Ulcer; iNOS, inducible Nitric Oxide Synthase; nor-NOHA, Nω-hydroxy-nor-arginine; VLU, Venous Leg Ulcer.

Over the last decades, there has been an increasing interest in the Arginase (ARG) pathways and their link to the proper progression through the wound healing cascade. ARG has been shown to have ample physiological implications, due to being the nexus of upstream signalling events as well as downstream metabolism of polyamines and proline (Caldwell et al., 2018). ARG dysfunction has been extensively linked to cardiovascular and neuropathic conditions (Gao et al., 2007; Pernow and Jung 2013; Caldwell et al., 2015). More and more studies are also highlighting a link between this enzyme and impaired wound healing (Jude et al., 1999; Abd-El-Aleem et al., 2000; Campbell et al., 2013; Abd El-Aleem et al., 2019). However, the implications of a malfunctioning ARG enzyme in wound chronicity are not well-understood.

This review aims to give an overview of ARG activity and the current understanding of how it relates to wound healing.

### Arginase Pathways Overview

ARG represents an evolutionarily conserved ureohydrolase enzyme involved in the final step of the urea cycle (Dzik 2014). This manganese (Mn)-containing metalloenzyme catalyses the conversion of L-arginine into L-ornithine and urea, thus being crucial for the disposal of toxic nitrogen resulting from amino acid (AA) and nucleotide metabolism (Meijer et al., 1990; Wu and Morris 1998).

The expression and activity of the enzyme ARG has been associated with several intracellular signalling events such as the activation of Rho kinase, Mitogen-Activated Protein Kinase (MAPK) and Protein Kinase A (PKA) (Shatanawi et al., 2011; Chandra et al., 2012). Moreover, multiple wound healing-related cytokines like Transforming Growth Factorβ (TGFβ), Interleukin-4 (IL-4), IL-6, IL-8, IL-13 and Tumour Necrosis Factor α (TNFα) have also been linked to the modulation of ARG activity, TGFβ being one of the most potent activators of ARG1 (Gordon 2003; Gao et al., 2007; Chandra et al., 2012). The Reactive Oxygen Species (ROS) produced early in the wound microenvironment might also modulate ARG activity, the ARG1 promoter region containing potential redox-sensitive elements (Kawamoto et al., 1987). ARG1 is also a CCAAT/Enhancer-Binding Protein-β (C/EBP-β) target gene (Ruffell et al., 2009). The activity of C/EBP-β is antagonised by Protein phosphatase 6 (Pp6), an epidermis-specific Ser/Thr phosphatase is essential for skin homeostasis (Lou et al., 2020).

#### Enzyme Isoforms and their Distribution

ARG has two isoforms ARG1 and ARG2, each one being encoded by different genes located on separate chromosomes. ARG1 is localised in the cytosol and, although it is mainly expressed in the liver and immune cell populations such as alternatively activated macrophages (AAMs) (Pauleau et al., 2004), it is also found in multiple cell types involved in the wound healing process including fibroblasts, keratinocytes and endothelial cells (Albina et al., 1990; Witte et al., 2002; Kampfer et al., 2003). Endothelial cells express both ARG isoforms (Buga et al., 1996; Bachetti et al., 2004), while smooth muscle cells possess ARG1 activity only (Durante et al., 1997). In fibroblasts ARG1 activity is upregulated by certain growth factors like TGFβ and haemodynamic forces including cyclic stretch (Durante et al., 2000).

ARG2 is present in the mitochondria and is highly expressed in tissues such as the kidneys, brain and retinas (Caldwell et al., 2018). ARG1 consists of 322 AAs (Dizikes et al., 1986), while ARG2 comprises 354 AAs (Gotoh et al., 1996). The two isoforms having a 60% homology in AA residues, whilst the areas critical to their catalytic function have 100% homology (Vockley et al., 1996).

**FIGURE 1:**
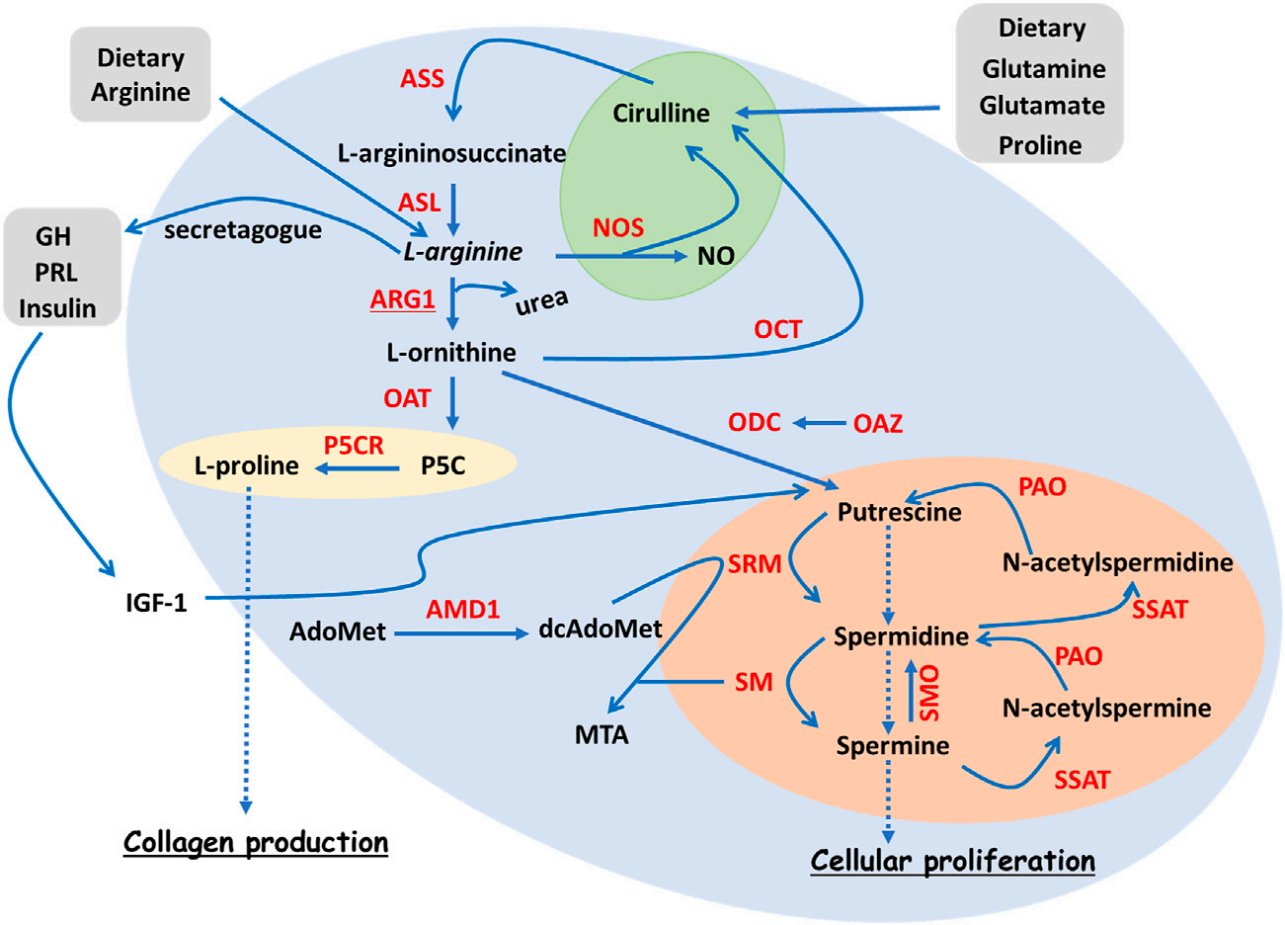
Overview of L-arginine metabolism. Simplified diagram summarising the ARG pathways, together with polyamine and collagen synthesis. The NOS branch of the ARG pathways is highlighted in green, polyamine production in orange and collagen production in yellow. L-arginine is an AA that can be derived either from diet or from recycled L-citrulline deriving from glutamate, glutamine or proline. L-arginine can be metabolised by ARG1 during the urea cycle, L-ornithine and urea being its breakdown products. L-ornithine, in turn, can be the precursor of several molecular events including the synthesis of L-proline via OAT, the synthesis of the polyamine putrescine *via* ODC, and the production of L-citrulline *via* OCT. L-proline represents a key collagen building block. Putrescine, together with its downstream products spermidine and spermine, make up the reversible polyamine pathway that contributes to cell proliferation. An important rate-limiting enzyme of the polyamine pathway is AMD1. L-arginine is also a secretagogue for growth hormone (GH), which contributes to cell proliferation *via* IGF-1-dependent polyamine pathway enhancement. Finally, L-arginine can be metabolised by NOS, the reaction products being NO and L-citrulline. Enzymes are highlighted in red. Abbreviations: AA, amino acid; AdoMet, S-Adenosyl methionine; AMD1, AdoMet decarboxylase; ASL, argininosuccinate lyase; ASS, argininosuccinate synthase; dcAdoMet, decarboxylated AdoMet; GH, growth hormone; IGF-1, insulin-like growth factor 1; MTA, 5′-methylthioadenosine; NO, nitric oxide; NOS, nitric oxide synthase; OAT, ornithine aminotransferase; OAZ, ornithine decarboxylase antizyme; OCT, ornithine carbamoyl transferase; ODC, ornithine decarboxylase; P5C, L-∆1-pyrroline-5-carboxylate; P5CR, P5C reductase; PAO, polyamine oxidase; PRL, prolactin; SM, spermine synthase; SMO, spermine oxidase; SRM, spermidine synthase; SSAT, spermidine ⁄ spermine N1 -acetyltransferase.

As illustrated in Figure 1, Nitric Oxide Synthase (NOS), the antagonist of ARG, is an enzyme catalysing the conversion of L-arginine into L-citrulline and Nitric Oxide (NO) (Schwentker and Billiar 2003). There are three different isoforms of NOS with slightly different functions and cellular localisation, namely neuronal NOS (nNOS or NOS1), inducible NOS (iNOS or NOS2) and endothelial NOS (eNOS or NOS3) (Witte and Barbul 2002; Schwentker and Billiar 2003).

Whilst nNOS and eNOS are discretely expressed in the brain and blood vessels respectively, iNOS is located in a variety of tissues, including the skin (Frank et al., 2002; Nieves and Langkamp-Henken 2002; Witte and Barbul 2003). Both eNOS and nNOS represent Ca^2+^-dependent isoforms that are constitutively expressed in their specific tissues (Nathan and Xie 1994), whilst iNOS is Ca^2+^-independent and mainly produced in pathological conditions (Ischiropoulos et al., 1992). This review will mainly focus on the functions of the iNOS and eNOS isoforms.

#### L-arginine in ARG and NOS Homeostatic Push-And-Pull

L-arginine is a dibasic, conditionally non-essential AA derived from either protein turnover or diet (Figure 1). This AA is tightly linked to several metabolic pathways involved in the synthesis of creatine, agmatine, urea, nitric oxide (NO) and polyamines (Witte and Barbul 2003). Decreased L-arginine plasma levels are observed in patients with trauma, burn wounds or those undergoing surgery, this reduction in its availability being associated with the increased metabolic demand triggered by the systemic inflammatory responses (Nijveldt et al., 2000; Ochoa et al., 2001; Yu et al., 2001). Randomised clinical trials in patients with severe skin burns highlight the beneficial effects of L-arginine enteral administration on burn healing, further suggesting a necessary increase in L-arginine catabolism (Yan et al., 2007). This increased L-arginine metabolism in the context of skin wounds is a first clue to the implications of ARG pathway in wound healing.

L-arginine represents the common substrate that ARG and NOS compete for (Figure 1). Therefore, under homeostatic conditions, the activities of these two enzyme systems are mutually exclusive. Certain regulatory mechanisms account for further activity control; Nitrite (NO_2_), the stable metabolite of NO, suppresses ARG (Hrabak et al., 1996), while the polyamine spermidine, an ARG downstream metabolite, can suppress NOS activity (Szabo et al., 1994).

#### ARG Pathway and Polyamine Production

Due to being a secretagogue for Growth Hormone (GH) (Nieves and Langkamp-Henken 2002), L-arginine indirectly induces the secretion of Insulin-like Growth Factor-1 (IGF-1) (Merimee et al., 1965; Merimee et al., 1967). IGF-1, which is also important for cell growth, acts, in turn, on Ornithine Decarboxylase (ODC; Figure 1) which is the first rate-limiting enzyme involved in polyamine synthesis. ODC decarboxylates L-ornithine into putrescine (Regunathan and Reis 2000; Witte and Barbul 2003). Putrescine is then sequentially converted into spermidine and spermine through the addition of an aminopropyl group from dcAdoMet. dcAdoMet results from the decarboxylation of AdoMet *via* Adenosylmethionine Decarboxylase 1 (AMD1), the second rate-limiting enzyme of the polyamine pathway (Figure 1) (Casero et al., 2018).

The polyamines are important for cell growth and proliferation (Langkamp-Henken et al., 1998; Li et al., 2001), being able to modulate a wide range of cellular functions including transcription, translation, cytoskeleton assembly and ion transport (Iarashi and Kashiwagi 2010; Lightfoot and Hall 2014).

#### ARG, Collagen Production and Fibrosis

Besides polyamines, L-ornithine is also the precursor for proline, the AA produced through the activity of Ornithine Aminotransferase (OAT; Figure 1). Proline is one of the main constituents of collagen and its increased production contributes to the post-traumatic increase in wound tensile strength, parenteral administration of L-arginine enhancing wound hydroxyproline levels in the dorsal skin incisions of both control and diabetic Lewis rats (Witte and Barbul 2003; Caldwell et al., 2018). Enhanced collagen production can lead to connective tissue thickening and subsequent fibrosis, as shown by studies based on both the activity of proline hydroxylase and the collagen incorporation rate of radiolabelled proline in skin tissue explants (Craig 1975; Rockwell et al., 1989). The enzymatic activity of proline hydroxylase correlates well with the rate of collagen synthesis, and the activity of this enzyme is markedly increased in the fibrotic areas of hypertrophic and keloid scars, when compared to normal scars (Craig 1975), thus putatively linking an enhanced collagen synthesis to the aetiology of fibrosis. The collagen synthesis rate was determined to be 3 times higher in hypertrophic scars and 20 times higher in keloids, when compared to normal scars (Rockwell et al., 1989). Fibrotic areas can also be seen in the dermal tissue surrounding chronic wounds (Abd-El-Aleem et al., 2000; Eming et al., 2014).

Wound healing represents an intricate multifactorial process whose slight dysregulations can impair its overall success. Recent studies have implicated the ARG pathway in several wound healing-related cell types and stages with macrophages being the most extensively studied (Campbell et al., 2013). However, given its wide expression in the wound site it is likely that arginase in cell types such as fibroblasts and keratinocytes will play an important role in healing and their contribution is less well-defined but should not be underestimated. Irrespective of the sources of ARG in the wound it is important to consider the impact of ARG on would healing. The following sections will review the involvement of ARG in key stages of wound healing, while emphasising the current understanding of the link between the ARG pathways and skin healing impairments.

#### ARG and iNOS in acute Wound Healing

In acute wounds, iNOS activity predominates in the early inflammatory stage where the wound microenvironment is cytotoxic. Infiltrating inflammatory cells such as macrophages and neutrophils synthesize large amounts of NO (Lyons et al., 1992). The effects of NO release, including anti-microbial activity (Granger et al., 1988), vasodilation (Stuehr et al., 1989), and anti-platelet aggregation activity (Salvemini et al., 1989), are important for the initial wound decontamination and debridement.

In the late healing stages, iNOS levels return to normal and ARG activity predominates (Albina et al., 1990), leading to the production of proline for collagen, and polyamines which are essential for cell growth, differentiation and matrix modelling (Sarhan et al., 1992; Shearer et al., 1997). Intracellular polyamine levels increase in response to cellular damage, this increase highlighting their putative role in the proliferative stage of wound healing (Casero and Pegg 2009; Gao et al., 2013). Moreover, a change in the putrescine/spermine ratio is important for epithelial tongue migration and wound re-epithelialisation *via* Urokinase-type Plasminogen Activator (uPA)/uPA receptor (uPAR)-driven actin cytoskeleton reorganisation (Lim et al., 2018).

Due to the complexity of the wound healing phases, the variable skin architecture of the outbred human population and differing microbial residency, impairments of skin-related molecular/cellular mechanisms can arise, leading to the occurrence of chronic wounds. The next sections are focused on the implication of the ARG pathways in the pathophysiology of chronic skin conditions, including ulcers and infected chronic wounds. Attention is given to the role that ARG plays in key cells of the wound healing process such as macrophages and fibroblasts. Current ARG pathway-based chronic wound treatments are also described, and future perspectives are emphasised.

## Macrophages, ARG Pathway and Wound Healing

Macrophages represent a crucial immune cell population with extensive physiological implications, among which is the regulation of wound healing progression. The importance of macrophages in the wound microenvironment is going to be detailed in the next section, emphasis being given to the activity of the ARG pathway in this cell population.

### Macrophages as Master Regulators of Wound Healing

Whilst macrophages seem to be dispensable in early foetal wounds (Martin et al., 2003), several murine knockout models have highlighted the need for macrophages in normal adult wound healing (Nagaoka et al., 2000; Peters et al., 2005; Ishida et al., 2008).

Macrophages persist throughout the entire healing response, their numbers increasing during the inflammatory stage, peaking during the tissue formation phase and finally declining during the maturation step (Martin and Leibovich 2005). The use of Diphtheria Toxin (DT)-mediated macrophage depletion in Lysozyme M (LysM) Cre/inducible Diphtheria Toxin receptor (iDTR) mice at early, mid and late wound repair stages showed the importance of macrophage involvement throughout the entire healing process. Inflammatory phase-restricted macrophage depletion led to impaired granulation tissue formation and re-epithelialisation, while the mid proliferative stage-restricted macrophage ablation resulted in severe wound site haemorrhage (Lucas et al., 2010).

In light of their ubiquity and extensive functions, Lawrence et al*.* have described macrophages as the “orchestra leaders” of adult wound healing (Lawrence and Diegelmann 1994). Critical to the function of macrophages in the healing process is their plasticity.

### Macrophage Plasticity and ARG Pathway

As far as the implications of macrophage-related L-arginine metabolism are concerned, the initially high levels of wound site pro-inflammatory cytokines, such as Interferon γ (IFNγ), TNFα and IL-1β, stimulate the iNOS-dependent production of large amounts of NO by pro-inflammatory classically activated (CAM) or M1 macrophages (Witte and Barbul 2002). NO may then spontaneously react with O_2_^−^, thus yielding toxic peroxynitrous acids, peroxynitrite and hydroxyl (OH^−^) radicals involved in pathogen killing (Beckman et al., 1990; Radi et al., 1991; Jude et al., 1999; Goldman 2004). In contrast, macrophage stimulation via cytokines such as IL-4, IL-10, IL-13 and IL-33 leads to a tissue reparative alternatively activated (AAM) M2 macrophage phenotype (Locati et al., 2013; Wang et al., 2014) associated with an enhanced ARG1 expression and activity (Munder et al., 1998; Pauleau et al., 2004). This cytokine-based phenotype switch highlights the plasticity of these heterogeneous leukocytes and its dependency on their surrounding milieu (Modolell et al., 1995; Shapouri-Moghaddam et al., 2018).

Studies focusing on the dichotomous far ends of the macrophage phenotypic spectrum, namely CAMs/M1 and AAMs/M2, have suggested that local macrophage ARG1 activity is vital for proper cutaneous wound healing (Gordon and Taylor 2005; Mosser and Edwards 2008). Differentiated macrophages appear to have distinct roles in the healing process-the activity of iNOS predominating in the early stages of wound healing in CAMs followed by high ARG activity later in the healing process in AAMs (Campbell et al., 2013; Krzyszczyk et al., 2018). Moreover, both the local pharmacological inhibition of ARG using nor- N^ω^-hydroxy-L-arginine (nor-NOHA) (Tenu et al., 1999; Takahashi et al., 2010) and the Tie2-mediated conditional ablation of ARG1 (El Kasmi et al., 2008) triggered pronounced delays in wound healing, suggesting a possible link between chronic wounds, ARG dysfunction and macrophage phenotypic transition.

This phenotypic transition from a M1 pro-inflammatory to a M2 anti-inflammatory macrophage was manipulated by Kim et al*. via* an exosome-guided reprogramming of cell polarity, with the purpose of accelerating wound healing. The subcutaneous administration of high purity M2-exosomes into the excisional wound edge of 5-week-old male Balb/c mice led to better wound healing outcomes by means of a successful M1 to M2 phenotypic switch although only CD86, iNOS and CD206, ARG1 were used to distinguish M1 and M2 macrophages respectively and fuller characterisation was not done (Kim et al., 2019). Even if promising, the therapeutic viability of this *in situ* exosome-based phenotypical transition remains questionable as the study did not test their effectiveness in animal models with underlying pathological conditions. Moreover, a challenge in studying macrophage function includes the usage of iNOS and ARG1 as markers of M1 vs. M2 phenotypes, as certain macrophages (such as the alveolar macrophages) express both markers (Thomas and Mattila 2014). Mouse age may pose a further difficulty, especially from a translational perspective, the incidence of wound healing problems and chronicity increasing with age (Jackson et al., 2017).

Due to their pervasive role in the wound healing process, it becomes obvious that macrophage malfunctions are a likely contributor to the pathophysiology of chronic wound impairments. The next section will focus on the underlying causes of skin ulcers while highlighting the implication of ARG1 and macrophage dysfunction in impaired healing/chronicity.

### ARG1 in Chronic Wounds

Owing to its expression in several cell types involved in wound healing as well as its ability to influence cell proliferation and collagen deposition by means of its downstream metabolites (Caldwell et al., 2018), it can be assumed that ARG might play a role in the non-healing status of chronic wounds. Ulcers represent prevalent chronic lesions that arise as a result of halted progression through the wound healing cascade (Agren et al., 2000). Given that ARG is involved in wound healing and skin ulcers represent a sequela of impaired cutaneous healing, the possible relationship between a malfunctioning ARG pathway and the underlying aetiology of ulcers is important to consider.

Ulcers represent a major concern from both a clinical and economic point of view, as the number of patients suffering from chronic wounds is reaching epidemic proportions (Brem and Tomic-Canic 2007; Sen et al., 2009; Guest et al., 2018). Moreover, the aging, multi-morbid population poses a further burden to the clinical world, the management of ulcers having to cater for both the systemic conditions such as diabetes as well as the local wound disorders (Eming et al., 2014). Depending on the underlying causes, there are several types of ulcers (Eming et al., 2014). For the purpose of this review, Venous Leg Ulcers (VLUs) and Diabetic Foot Ulcers (DFU) will be the main focus. However, it is worth noting that Arterial Ulcers (AU) and Pressure Ulcers (PU) represent important clinical challenges as well (Eming et al., 2014).

VLUs represent a common form of leg ulcers with increased incidence among the elderly. VLUs are one of the most severe symptoms of low extremity chronic venous insufficiency (Bergan et al., 2006; Raju and Neglén 2009). DFUs are regarded as one of the most common reasons for hospital admission among diabetic patients (Williams 1985), leading to 50–70% of all non-traumatic amputations (Boulton 1997; Margolis et al., 2005). Even more worrying is the anticipation that the prevalence of Type 2 Diabetes Mellitus (T2DM) will increase from 6.4 to 8% in the world population by the year 2030 making the incidence of DFUs potentially even more common (Nolan et al., 2011).

### VLU and DFU Pathophysiology Overview

VLUs are characterised by venous hypertension, persistent inflammation, hemosiderin deposition and lipodermatosclerosis (Eming et al., 2014). This chronic inflammatory state suggests a failure of VLUs to progress through the physiological pattern of wound healing stages (Agren et al., 2000; Smith 2001; Wlaschek and Scharffetter-Kochanek 2005). The hallmarks of DFUs are hyperglycaemia, micro-/macroangiopathy, neuropathy and infection, the condition usually culminating in foot deformities (Eming et al., 2014). Similar to VLUs, diabetic wounds show an unresolved inflammatory state, suggesting a hindrance of the transition from the pro-inflammatory to the proliferative stage of wound healing (Sibbald and Woo 2008).

### Increased NOS Activity in Both VLUs and DFUs

In chronic venous ulcer histological sections, the expression of both eNOS and iNOS was increased when compared to normal skin samples (Shimizu et al., 1997; Abd-El-Aleem et al., 2000). This enhanced NOS expression correlated with an increase in NOS activity, suggesting a higher NO production than that of normal skin tissue (Abd-El-Aleem et al., 2000). In addition, a venous stasis-dependent accumulation of NOS-expressing M1 macrophages is one of the prevalent features of VLUs (Rosner et al., 1995; Abd-El-Aleem et al., 2000), with hypoxia and the low shear stress contributing to the transmigration of such “trapped” leukocytes into the tissue. Once there, they release high amounts of ROS such as Fenton reaction-derived OH^−^ (Yeoh-Ellerton and Stacey 2003), proteolytic enzymes like MMP2 or MMP9 (Wysocki et al., 1993), and proinflammatory cytokines such as TNFα, IL-1 and IL-6 (Tarnuzzer and Schultz 1996). These contribute to extensive connective tissue breakdown and subsequent ulcerations (Wlaschek and Scharffetter-Kochanek 2005). The large amounts of NO produced *via* iNOS activity interact with the ROS resulting from the respiratory burst of the macrophages/neutrophils present at the wound site, leading to the formation of peroxynitrite (Beckman et al., 1990). Both NO and peroxynitrite cause apoptotic cell death, thus contributing to the damage seen in chronic venous ulcers (Albina et al., 1993; Messmer et al., 1994; Lin et al., 1995). Moreover, Sindrilaru et al*.* have directly linked the persistent inflammation and ECM breakdown of VLUs with a perpetuated M1 proinflammatory state of iron-overloaded macrophages, as iron chelation, etanercept-dependent TNFα inhibition and clodronate-induced macrophage depletion improve the phenotype of these ulcers (Sindrilaru et al., 2011).

As far as diabetic ulcers are concerned, eNOS is increased at the base and edge of the ulcer in DFUs when compared to normal and diabetic human skin, thus showing similarities to the eNOS expression seen in VLUs (Table 1). In addition, an increased iNOS expression was noted at the ulcer margins, predominantly localised to the smooth muscle cells of the blood vessels as well as the infiltrated macrophages (Jude et al., 1999). The similarity between this iNOS localisation and that seen in VLUs provides a further commonality between these two ulcer types (Table 1). As well as expression of eNOS and iNOS, there is an increased total NOS activity in DFUs, when compared to normal and diabetic skin and the NOS is thought to be largely macrophage derived (Jude et al., 1999). Similar findings have been reported in rodent models (Stevens et al., 1997). Upregulated catalytic activity in DFU patients was also mirrored by increased plasma NO_2_ levels, and treatment with insulin lowered NO_2_ concentration in these sufferers (Stevens et al., 1997; Jude et al., 1999). The excessive levels of nitrite may provide a likely explanation for the DFU-associated neuropathies. The increased NOS activity of ulcers was further corroborated in a recent study by Dixit et al. conducted in 61 patients with chronic wound cases, DFUs and VLUs being the most predominant lesions. By comparing the tissue and serum enzyme activities at initial patient presentation and 12-weeks follow-ups, an increased expression and activity of NOS was noted at the second time point (Dixit et al., 2021). Thus, this further highlights the putative metabolic implications of the NOS isoforms in chronic wound healing.

**TABLE 1:** Summary of key ARG pathway-related pathological commonalities between DFUs and VLUs. Although NOS and ARG are known to have competitive enzymatic activities when expressed within the same cell, they both contribute to DFUs and VLUs, as they show discrete wound expression sites. iNOS is mainly present in endothelial cells and macrophages present at the ulcer base, contributing to defective matrix deposition and necrosis *via* excessive NO. ARG is mainly expressed in the dermal fibroblasts of the skin surrounding the ulcer itself. The increased ARG activity of both ulcer types is associated with fibrosis surrounding the ulcerations, resulting in either calluses or lipodermatosclerosis in DFUs and VLUs respectively. The enhanced levels of the cytokine TGF-β noted in the sclerotic skin adjacent to the venous ulcer further contributes to this excessive ARG activity.

Category	Feature	References
ARG activity and distribution	Hyperactive ARG in the fibroblasts, macrophages and endothelial cells of surrounding dermis leading to pathological fibrosis surrounding ARG-hindered ulceration	Herrick et al. (1992)
		Jude et al. (1999)
		Abd-El-Aleem et al. (2000)
NOS activity and distribution	Upregulated e/iNOS activity in wound bed M1 pro-inflammatory macrophages leading to large amounts of NO and subsequent necrosis/ulceration	Albina et al. (1993)
		Messmer et al. (1994)
		Lin et al. (1995)
		Jude et al. (1999)
Inflammatory status	Perpetuated M1 pro-inflammatory macrophages contributing to protracted inflammation *via* TNFα, IL1 and IL6 within ulceration, and hindering transition to ARG-related proliferative stage	Sindrilaru et al. (2011)
Expression and distribution of proteolytic enzymes	Excessive wound bed ECM breakdown due to abnormal levels of macrophage/neutrophil MMP2 and MMP9 overriding ARG-dependent ECM deposition	Herrick et al. (1997)
ROS and tissue oxidative stress	Excessive ROS-dependent DNA damage causing fibroblast senescence within wound bed and impairing ECM deposition, despite enhanced ARG activity	di Fagagna et al. (2003)
		Garinis et al. (2009)
Growth factors	High levels of ARG-inducing TGF-β in sclerotic skin adjacent to ulcer, but drastically reduced within ulcer per se	Higley et al. (1995)
		Boutard et al. (1995)
		Stevens et al. (1997)
Angiopathies	Perivascular cuffs and reduced compliance associated to proliferative and matrix deposition effects of excessive ARG activity	Hagenfeldt et al. (1989)
		Pieper and Dondlinger (1997)
		Kovamees et al. (2016)

### Increased ARG Activity in Both VLUs and DFUs

Perhaps surprisingly given the high levels of NOS, VLUs show increased ARG activity concentrated in fibroblast- and macrophage-like cells present in both the epidermis and the dermis surrounding the wound bed (Abd-El-Aleem et al., 2000; Sindrilaru et al. 2011)*.* However, these macrophages fail to fully switch from a pro-inflammatory M1 state to a tissue repair M2 one and transiently co-express high levels of M1 associated markers such as TNFα and C-C chemokine receptor type 2 (CCR2) as well as intermediate levels of M2 markers like ARG, CD36 and CD206 (Sindrilaru et al., 2011).

Despite the upregulation of ARG in chronic venous ulcers, the matrix deposition was defective. The upregulation of certain proteases like Ser proteases and neutrophil elastases might explain this discrepancy, as their excessive proteolytic activity leads to the breakdown of fibronectin within the wound bed (Herrick et al., 1997). The lack of a fibronectin scaffold for fibroblast integrin-dependent migration hinders their matrix deposition capabilities. In addition, the enhanced release of ROS, especially OH^−^ and peroxynitrite, by the macrophage population described by Sindrilaru et al*.* is a likely contributor to this defective matrix deposition. The extensive ROS-related DNA damage (Szabo et al., 2007) triggers a senescent program in skin resident fibroblasts (di Fagagna et al., 2003; Garinis et al., 2009), thus impairing their tissue repair capabilities (Sindrilaru et al., 2011). Higher ARG levels may, therefore, be explained, in part, as a consequence of a failed compensatory mechanism for the extensive wound bed tissue breakdown.

Akin to VLUs, ARG expression is high in dermal fibroblasts and endothelial cells (Jude et al., 1999, Table 1). This ARG upregulation was shown to be involved in the pathological fibrosis seen in the callus that surrounds the DFU (Jude et al., 1999) and has also been described in rodent studies (Kampfer et al., 2003; Miao et al., 2012). Similarly, significantly increased ARG has been reported in chronic skin wounds 12 weeks after initial presentation (Dixit et al., 2021). It is not fully known why these defective transitions occur in ulcers but one factor may be due to a lack of critical cytokines such as TGFβ1 in the wound environment.

### Lack of TGFβ as an Ulceration-Related Factor

When compared to both normal and diabetic skin, macrophage TGFβ1 expression in DFUs is drastically reduced or even absent (Stevens et al., 1997; Jude et al., 1999). Given that TGFβ increases the ARG activity at the expense of NOS activity (Boutard et al., 1995), this lack of TGFβ1 in the ulcer macrophages translates into an impaired transition from the tissue debridement to the tissue repair stage. This provides a possible reason for the NO-rich necrotic area characteristic of DFUs (Ferguson et al., 1996), a raised and sustained NOS activity being deleterious. Moreover, the diabetes-specific hyperglycaemic environment impairs the normal phenotypic transition of wound macrophages as well as their iNOS/ARG activity with compromised responses to their instructive cytokines being noted (Gordon 2003; Gordon and Taylor 2005; Miao et al., 2012). However, much remains to be learnt about factors involved in driving macrophage polarisation with the chronic wound bed.

### Systemic Complications of Excessive Ulcer ARG1 Activity

The high levels of ARG1 noted in several cell types present within and around the granulation tissue of both VLU and DFUs, including fibroblasts, macrophages, endothelial and vascular smooth muscle cells, might contribute to systemic complications as well. A polyamine-driven hyperplasia or hypertrophy of blood vessels may lead to the underlying venous hypertension characteristic of VLU patients. What is more, the ARG1-triggered excessive collagen deposition further contributes to this hypertensive phenotype, due to the thickening and stiffening of blood vessels (Table 1). These blood vessel architecture-related assumptions are corroborated by studies showing that coronary arteries isolated from diabetes patients had increased ARG1 expression and exhibited an impaired endothelium-dependent vasorelaxation (Bagi et al., 2013). Furthermore, other studies highlighted a correlation between increased ARG activity, decreased plasma L-arginine levels and endothelial dysfunction in type 2 diabetic patients and diabetic rats, reinforcing the idea of ARG-dependent pathological vascular remodelling, fibrosis and decreased compliance in DFUs (Table 1) (Hagenfeldt et al., 1989; Pieper and Dondlinger 1997; Kovamees et al., 2016). However, this hyperactive ARG does not help with tissue deposition and repair within the wound per se.

This section emphasises the possible implications of ARG in ulcers at both local and systemic levels while highlighting the similarities between DFUs and VLUs. This suggests that there might be a certain molecular common ground between these two ulcer types which would be worth exploiting when it comes to therapeutical options. Indeed studies have highlighted the etiological commonalities between different ulcerations (Chen and Rogers 2007).

In addition to the putative implications of ARG in conditions like ulcers, its activity is also associated with the balance micro-organismal populations residing on the skin. Skin infection in chronic wounds is a major risk factor in poor healing. Therefore, the next section studies the implications of ARG in the context of chronic infected wounds.

## ARG and the Skin Microbiota

The microbial composition of wounds also play an important role in the healing process and alterations in skin bacterial communities have been associated with VLUs and DFUs, among other skin conditions (Hannigan and Grice 2013; Weyrich et al., 2015). ARG activity has been linked to the presence of certain pathogens within the wound microenvironment, further suggesting the far-reaching implications of this enzyme in wound healing (Debats et al., 2006). This section will explore the changes seen in wound-related microbiota, as opposed to normal skin microorganisms, and how these impact on the activity of the ARG pathway and vice versa.

### Homeostatic and Wound-Related Skin Microbiota

The skin is heavily populated by bacteria, viruses, archaea and fungi, all contributing to the holobiont. There is a constant crosstalk between the skin microorganism and the host as changes in the microbial communities populating the skin can trigger the expression of antimicrobial peptides and inflammatory molecules (Zeeuwen et al., 2012). Even in the absence of overt signs of infection, the impairment seen in certain chronic wounds has been associated with the presence of a specific bioburden consisting of bacteria belonging to the *Staphylococcus, Pseudomonas, Peptoniphilus, Enterobacter, Stenotrophomonas, Finegoldia, and Serratia* genera (Gardner et al., 2013; Eming et al., 2014; Misic et al., 2014; Rahim et al., 2017). These polymicrobial communities exist mainly in the form of tightly packed aggregates with synergistic virulence known as biofilms (James et al., 2008). The biofilm composition differs from that of normal skin, the latter consisting mainly of resident flora such as *S. epidermidis* or *Propionibacterium*, and transient flora such as *S. aureus* or *Proteus mirabilis*, different body parts having varying micro-organismal proportions or abundances (Rahim et al., 2017).

### Hallmarks of Infected Chronic Wounds and Impact on L-arginine Metabolism

According to a comparative study between patients displaying acute, chronic non-infected and chronic infected wounds, acute wounds have considerably lower indicators of inflammation and a lack of infection signs, with no bacteria cultured from their wound fluid (Debats et al., 2006) whereas infected chronic wounds are often associated with *Pseudomonas aeruginosa*, *Staphylococcus aureus*, *Escherichia coli*, and *Proteus mirabillis* (Debats et al., 2006). Considerably decreased levels of plasma L-arginine were noted in patients with infected chronic wounds, when compared with both acute and non-infected chronic wound patients (Debats et al., 2006). Despite decreased plasma L-arginine, no differences in systemic inflammatory markers such as Erythrocyte Sedimentation Rate (ESR) and albumin were noted when infected chronic wounds were compared with their non-infected counterparts (Debats et al., 2006) indicative of a local rather than a systemic response. Therefore, this suggests an increased uptake and metabolism of L-arginine at the site of chronic infected lesions, the infection being the likely driver of this increased metabolic activity. However, it should be pointed out that both ESR and albumin are crude, non-specific markers of systemic inflammation, certain endogenous pyrogens, such as IL-6 and TNFα, representing better options.

Wound fluid L-citrulline, L-ornithine and ARG1 levels are significantly increased in infected chronic wounds, when compared to non-infected wounds and normal skin (Debats et al., 2006). These increased levels of certain AA at the wound site may be explained by an extensive proteolysis. However, L-ornithine is a non-proteinogenic AA and, hence, it is not incorporated into polypeptide chains during protein biosynthesis. Therefore, it is unlikely that L-ornithine is released by proteolysis. This seems to suggests that the increase in wound fluid L-ornithine is the likely consequence of an increased ARG1 activity (Debats et al., 2006). Although the synthesis and breakdown of L-ornithine are important for producing wound healing-related metabolites (Figure 1) (Bachrach et al., 2001; Urdiales et al., 2001), these excessive L-ornithine levels paradoxically contribute to wound chronicity rather than accelerated wound closure.

Significantly decreased levels of the NO metabolites nitrite and nitrate were noted in infected chronic wounds, while their plasma levels were similar to that of acute and non-infected chronic lesions (Debats et al., 2006). This, together with enhanced ARG levels, strongly suggests that the NOS activity within the chronic infected wound is reduced. However, the increase in wound citrulline production then seems paradoxical. An explanation for the unexpected increase in citrulline is provided by the presence of Arginine Deiminase (ADI)-expressing bacteria like *P. aeruginosa* and *E. coli*. This enzyme can catalyse the direct conversion of L-arginine into citrulline (Galkin et al., 2004; Debats et al., 2006). Indeed, when cultured in the fluid derived from infected wounds, these bacteria use L-arginine in order to generate energy in the form of ATP (Theil et al., 1969; Lu et al., 1999), therefore the inherent bacterial catalytic ability may account for overall increased wound citrulline. Moreover, the decreased production of bactericidal NO may further contribute to defective barrier function in infected wounds where microorganism growth is not being hindered by a NO-rich microenvironment. This seems to suggest that the excessive ARG activity may contribute to bacterial growth and thus impaired wound healing of infected lesions. However, macrophage ARG1 activity has been shown to play a central role in restricting bacterial growth and restraining tissue damage in hypoxic tuberculosis granulomas (Duque-Correa et al., 2014), suggesting that the relationship between ARG activity and bacterial burden might be tissue- and pathology-specific.

In light of what has been said, the presence of an infection is a likely contributor to the hindered progression of the wound healing process and it is notable that successful wound closure is associated with a low microbial count (Edwards and Harding 2004). Nevertheless, it is worth saying that certain studies emphasise the importance of bacterial colonisation, setting a threshold of 10^4^ Colony Forming Units (CFUs)/ml as a delineation between colonisation and wound healing-impeding infection (Trengove et al., 1996). Moreover, isolates from different parts of the same wound house different organisms (Schneider et al., 1983). Furthermore, the stability of the chronic wound microbiota is important as successful healing is associated with a flexible dynamic microbiota (Loesche et al., 2017).

Owing to the intricate interplay between molecular, cellular and microbial components associated with impaired wound healing, the difficulty of devising highly effective chronic wound treatments becomes obvious. The following section highlights some of the most common chronic wound therapeutical approaches based on the modulation of the ARG pathway.

## ARG Pathway-Based Chronic Wound Treatment Options

Given the far-reaching implications of the ARG pathway in wound healing and pathology as well as the extensive web of molecular components contributing to this pathway, the search for its druggable targets seems to be a sensible step in the attempt to design a multimodal treatment for chronic skin conditions. While some treatments focus on the AAs of this pathway by means of nutritional or parenteral supplementation, other current monotherapies aim to target the enzymatic nodes of the ARG pathway via pharmacological modulation. Both of these approaches will be discussed in this section.

### Nutritional Therapy

Nutritional factors have a crucial role in the development of chronic wounds (Schaffer et al., 1997). Nutritional therapy involving the intake of vitamins A and C (MacKay and Miller 2003) or proteins (Ruberg 1984; Breslow et al., 1993) in malnourished patients with chronic wounds has led to an improvement in wound healing, although complete wound closure was not observed (Thomas 2001; Houwing et al., 2003). A more targeted strategy proposes using the supplementation of specific AAs known to be of great importance in the wound healing cascade of events (Angele et al., 2002; Witte et al., 2002; Shi et al., 2003) such as L-arginine and L-Ornithine (Barbul et al., 1990; Albina et al., 1993; Efron and Barbul 2000). Unfortunately, little is known about the effects of the addition of exogenous L-arginine to chronic wounds, as to date enteral L-arginine supplementation was mainly studied in acute artificially induced wounds, in both humans and rodents. Parenteral means of administration have also been studied, intravenous, intraperitoneal, subcutaneous and dressing-based topical applications of L-arginine being the most common approaches. Both enteral and parenteral supplementation contributed to increased collagen synthesis and enhanced wound breaking strength (Stechmiller et al., 2005).

L-arginine supplementation has been associated with enhanced production of NO at the chronic wound site (Norris et al., 1995; Schaffer et al., 1996; Pollock et al., 2001) which may then contribute to granulation tissue formation, immune responsiveness and angiogenesis (Yamasaki et al., 1998; Schaffer et al., 1999; Witte and Barbul, 2002). Furthermore, L-arginine supplementation has been regarded as an adjunct treatment for either normalising or enhancing wound healing, since the body does not produce sufficient amounts of this AA during metabolically-demanding times of stress (Albina et al., 1988; Witte and Barbul 2003). Proper levels of L-ornithine are crucial for the production of proline (Albina et al., 1993), an essential AA for collagen synthesis (Morris 1992), and polyamines, key players in cell growth and proliferation (Dolynchuk et al., 1994; Wu and Morris 1998). L-ornithine may also regulate the catalytic activity of ARG via a feedback loop mechanism as high AA levels could be an inhibitor of ARG activity (Hunter and Downs 1945). Dietary polyamines should not be excluded as a treatment option either, as systemic administration of spermidine led to accelerated skin wound healing in a mouse excisional back wound model (Ito et al., 2021).

ARG-promoting treatments might also be worth considering. A biomaterial-based controlled release of IL-4 prompted a switch from M1 to ARG1-expressing M2 phenotype in RAW 264.7 macrophages, while the treatment of the same macrophage cell line with Echinacea-derived alcohol extracts enhanced their ARG activity (Zhai et al., 2009; Yang et al., 2018). These treatments, even though restricted to macrophage alternative activation and the enhancement of their ARG enzymatic activity, might prove to have overarching anti-inflammatory implications in the overall chronic wound healing outcome. However much remains to be elucidated about arginase modulation or its downstream metabolites supplementation in the healing response.

### Pharmacological Antagonism of ARG Activity

Pharmacological inhibitors of ARG or NOS represent other therapeutic means intended for targeting the activity of ARG itself. DFMO, a potent inhibitor of OCD, acts as a weak, nonspecific inhibitor of ARG (Table 2) (Morris 2009). The ARG inhibitory effect of the DFMO treatment can be mediated by the accumulation of L-ornithine feedbacking on ARG and limiting its activity (Hunter and Downs 1945; Caldwell et al., 2018). The potent inhibition of OCD by DFMO would also limit the polyamine production, this limitation preventing the backward metabolism of spermidine/spermine by PAO (Figure 1), therefore reducing the oxidative stress of the wound site (Caldwell et al., 2018). Two potent pharmacological inhibitors of ARG are NOHA and its analogue nor-NOHA which has a longer half-life (Table 2). However, the shortcoming of NOHA is that it is an intermediate metabolite in the NOS-mediated pathway of L-arginine breakdown (Caldwell et al., 2018).

**TABLE 2:** Summary of key ARG-pathway related drug pharmacodynamics. The inhibitory mechanisms of ARG pathway-targeting drugs are given together with their inhibition constants (Ki). * iNOS-specific range.

Drug	Pharmacology	K_i_	References
ABH	commercially available for preclinical use; highly specific competitive ARG inhibitor; its boronic head binds to the catalytic Mn(II) cluster of ARG	0.25 μM	Baggio et al. (1999)
			Colleluori and Ash (2001)
			Berkowitz et al. (2003)
			Caldwell et al. (2018)
AG	selective irreversible inhibitor of cytokine-induced iNOS; L-arginine analogue competitive at substrate and gene level	NA	Salinas-Carmona et al. (2020)
BEC	commercially available for preclinical use; highly selective competitive ARG inhibitor; its boronic head binds to the catalytic Mn(II) cluster of ARG	0.31 μM	Baggio et al. (1999)
			Colleluori and Ash (2001)
			Berkowitz et al. (2003)
			Caldwell et al. (2018)
DFMO	weak indirect inhibitor of ARG via potent ODC inhibition and L-ornithine accumulation feedback	3.9 mM	Selamnia et al. (1998)
			Morris (2009)
L-NAME	non-selective competitive reversible NOS inhibitor	4–65 μM *	Rees et al. (1990)
			Furfine et al. (1993)
NOHA	potent competitive endogenous ARG inhibitor; L-arginine analogue binding the ARG Mn(II) cluster; also acts as an intermediate in NOS-dependent L-citrulline production, thus being a bisubstrate	10 μM	Daghigh et al. (1994)
			Custot et al. (1997)
			Caldwell et al. (2018)
nor-NOHA	commercially available for preclinical use; reversible selective ARG inhibitor; more potent than its longer NOHA analogue; binds ARG Mn(II) cluster	0.5 μM	Custot et al. (1997)
			Caldwell et al. (2018)
SC-842	selective iNOS inhibitor; no further details found in literature	NA	Bell et al. (2007)

Competitive ARG inhibitors with high specificity have been developed, their design being based on the determination of the crystal structure of human ARG1/2 (Kanyo et al., 1996; Di Costanzo et al., 2005). These highly selective ARG inhibitors are S-(2-boronoethyl)-L-cysteine (BEC) and 2(S)-amino-6-boronohexanoic acid (ABH) (Baggio et al., 1999) and their strong competitive inhibition results from the binding of their boronic heads to the Mn cluster found at the catalytic site of ARG (Table 2) (Berkowitz et al., 2003). Despite their specificity for ARG, the drawback of using these pharmacological inhibitors is that they are not isoform-selective (Caldwell et al., 2018). Furthermore, there may be unintended consequences of blocking arginase indiscriminately as it expressed by so many cell types in the skin. The proper design of isotype-selective, cell-specific inhibitors may facilitate the future development of strategies aimed at modulating the enzymatic activity of ARG in order to restore its homeostatic activity in pathological conditions.

Of the ARG inhibitors described in this section, nor-NOHA, BEC and ABH are commercially available and have been used in preclinical animal models as well as in humans with promising results reported for the small-scale treatment of patients with hypertension (Holowatz and Kenney 2007) and T2DM (Shemyakin et al., 2012), however, there is no information currently available on any clinical trials in patients with chronic wounds.

Chronic wounds were also associated with a sustained upregulation of iNOS (Abd El-Aleem et al., 2000). Therefore, targeting this enzyme of the ARG pathways might also represent a therapeutic option. Given the known homeostatic push-and-pull mechanism between iNOS and ARG in the context of acute wound healing, it would be expected that iNOS inhibition might trigger the pro-healing activity of ARG. However, an *in vivo* study of incisional skin wound healing in hairless but immunocompetent SKH-1 female mice showed no difference in wound tensile strength or histopathological features (such as epidermal hyperplasia, fibrin deposition and oedema) upon iNOS inhibition *via* SC-842 (Bell et al., 2007). Moreover, iNOS inhibition by means of both selective (aminoguanidine; AG; Table 2) and non-selective (N-Nitro-L-Arginine Methyl Ester; L-NAME; Table 2) inhibitors delayed wound closure and increased scarring in an *in vivo* acute incisional back wound model involving adult male, Sprague Dawley rats (Abd El-Aleem et al., 2020).

### Natural Products with Wound Healing Properties

In addition to conventional drug-based treatments to specifically target ARG pathways, there is increasing interest in the use of traditional medicines as adjunctive therapies for chronic skin conditions (Kulprachakarn et al., 2017), and it is possible that these will modulate ARG pathways. Further study and validation are needed to understand the underlying mechanisms involved in their restorative effects. Notably, enhanced wound healing has been linked to the bioactive constituents of organic products such as *Achillea asiatica* (Dorjsembe et al., 2017), *Aloe vera* (Daburkar et al., 2014), catechins (Kapoor et al., 2004), grapefruit (Kandhare et al., 2014), honey (Vijaya and Nishteswar 2012) and turmeric (Ibrahim et al., 2018). The wound-healing related attributes of these constitutes include antibacterial, anti-inflammatory, antioxidant, and pro-collagen synthesis properties (Ibrahim et al., 2018). Given these attributes are closely linked to the effector functions of ARG, the possibility of their interaction with members of the ARG pathways cannot be excluded. Indeed, *in vivo* model studies of *Aloe vera*, grapefruit extracts (Kulprachakarn et al., 2017) and the catechin epicatechin galatte (ECG) (Kapoor et al., 2004) showed that there was modulation of the ARG pathways. A significant upregulation of iNOS activity was noted in the early stages of the wound healing process (days 1–3), whilst a significant decrease in ARG1 activity was observed during the same time interval (Kapoor et al., 2004). The applicability of natural compounds in clinical practice has seen an improvement in recent years, due to being incorporated into different formulations based on hydrogels, micelles or nanoparticles (Ibrahim et al., 2018). These formulations have helped overcome pharmacodynamic problems such as poor aqueous solubility and fast degradation rates, therefore enhancing medicine delivery and bioavailability (Ibrahim et al., 2018; Kant et al., 2021). As more of these products become more pharmacokinetically available, further work may assess and target the molecular components of the ARG pathways.

Even though several current options for the ARG-dependent treatment of chronic wounds and their pitfalls have been presented in this section, the fast-paced development of new technologies as well as our ever-improving understanding of the chronic wound aetiology should help us develop better preclinical and patient-related procedures in the near future. In clinical practice, chronic wound monotherapies have led to unsatisfactory results. Thus, the need for the design of a multimodal treatment for chronic skin conditions is imperative.

## Discussion, Future Options and Perspectives

The activity of ARG may be modulated by intrinsic differences in human skin. ARG activity in the context of wound healing may vary depending on the sex, age and demographics of the patients (Wong et al., 2016). Sex-related differences are important, as skin is thinner in women than men (Sandby-Møller et al., 2003) which is further exacerbated during the menopause due to depletion of oestrogen (Chen et al., 2001). Age differences between patients should also be taken into consideration, as the diminished elasticity, xerosis and immunosenescence noted in aging skin can have a considerable impact on the outcome of studies involving the ARG pathway (Leyden 1990).

In patients with chronic wounds, underlying systemic factors such as advanced age, compromised immunological status and constant mechanical stress can contribute to poor healing, as is the case of the ulcers described in this review. Due to the need to target both systemic comorbidities, like diabetes or hypoperfusion, and local/regional skin impairments, Eming et al. raise the question of possible drug-drug interactions in patients with chronic wounds. Therefore, the best treatment approach would be to normalise the underlying systemic condition while administering local treatment, paying close attention to the pharmacodynamics of the multiple drugs used (Eming et al., 2014). In addition, given that the chronic wound aetiology is multifactorial, a synergising multimodal treatment targeting several growth factors, ECM components and cell types, rather than the ARG pathway alone, may improve the quality of future treatments (Eming et al., 2014). As far as prospective clinical trials are concerned, their large-scale successfulness may depend on the improvement of inclusion criteria, proper patient stratification and standardisation. These factors may also facilitate the implementation and interpretation of future meta-analyses.

While ulcers highlight the likely implications of a malfunctioning ARG pathway in macrophages and fibroblasts, the pathological activity of ARG in other wound healing-related cells known to express this enzyme should not be neglected either. Due to inappropriate re-epithelialisation, the aetiology of chronic skin conditions has been strongly linked to keratinocytes. However, little attention has been given to the implications of ARG-expressing epidermal cells in the context of chronic wounds. Since the downstream metabolites of the ARG pathway (especially the polyamines) have been extensively associated with cell proliferation and differentiation, the links between the ARG pathway, the polyamines and inappropriate re-epithelialisation might be worth highlighting in the context of chronic wounds. Therefore, this represents a future research avenue that may improve our understanding of ARG in the context of non-healing wounds.

## Conclusion

To sum up, the ARG pathway is important in wound healing with multiple roles as discrete points of the healing cascade. Defects in arginase signalling, expression and/or function are common in delayed healing wounds. However, more must be known about its cell specific roles and how different morbidities and factors such as age and sex impact it. Indeed, the translational potential of emerging therapies aiming to fill the bench-to-bedside gap in delivering arginase linked therapies will likely depend on a better understanding of patient heterogeneity and stratification criteria. Nevertheless, manipulation of the arginase pathway is an exciting and important area to consider targeting to enhance healing and improve patient outcomes.
